# Physicochemical and antibiofilm properties of new ready-to-use and powder/gel bioceramic endodontic sealers

**DOI:** 10.1590/1807-3107bor-2025.vol39.076

**Published:** 2025-07-07

**Authors:** Jéssica Arielli PRADELLI, José Leandro de Abreu JAMPANI, Airton Oliveira SANTOS-JUNIOR, Mario TANOMARU-FILHO, Juliane Maria GUERREIRO-TANOMARU

**Affiliations:** (a) Universidade Estadual Paulista – Unesp, Araraquara School of Dentistry, Department of Restorative Dentistry, Department of Restorative Dentistry, Araraquara, SP, Brazil.

**Keywords:** Endodontics, Root Canal Filling Materials, Root Canal Obturation, Silicate Cement

## Abstract

NeoSealer Flo (NSF) is a novel ready-to-use endodontic bioceramic sealer, and NeoMTA2 (NMTA2) is a powder-gel bioceramic used as a repair or filling material. Setting time (ST), pH, solubility, radiopacity, and flow were evaluated in accordance with the ISO 6876:2012 standards for NSF and NMTA2, in comparison with Bio-C Sealer (BCS) and AH Plus Jet (AHP). Bond strength was assessed using the push-out test. Antibiofilm activity was evaluated by a modified direct contact test on *Enterococcus faecalis* biofilm with fresh sealer eluate and material after setting. Data were analyzed using the t-test, ANOVA, and Tukey post-hoc tests (α = 0.05). AHP exhibited the highest ST value, followed by NMTA2 (p < 0.05). NSF and BCS showed solubility above 3%, while NMTA2 exhibited values lower than 3% (p < 0.05). All materials demonstrated radiopacity above 3 mm Al (p > 0.05). NMTA2 demonstrated flow below ISO-recommendation (p < 0.05). NSF showed the highest bond strength values (p < 0.05). The bioceramic sealers induced higher pH compared with AHP (p < 0.05). NMTA2 exhibited more significant antibiofilm activity after the sealer set (p < 0.05). For fresh sealer eluate, NSF, BCS, and AHP demonstrated higher levels of antibiofilm activity compared with NMTA2 (p < 0.05). NSF and NMTA2 demonstrated the required physicochemical and mechanical properties, as well as antibiofilm activity against *E. faecalis*. However, NSF and BCS presented higher solubility. NMTA2 presented adequate solubility, however lower flow rates than those recommended by ISO standards.

## Introduction

Bioceramic sealers exhibit biocompatibility^1^ and bioactivity^2^ in addition to radiopacity and flow.^3^ These sealers release hydroxyl ions, promoting an alkaline pH^3^, capable of contributing to antimicrobial activity against persistent infections.^4^ Calcium silicate-based sealers must demonstrate adequate bond strength to dentin to reduce the interface between the material and root canal walls.^5^ However, bioceramic sealers may have solubility above the 3% limit recommended by the ISO 6876:2012 standard.^3^

NeoSealer Flo (NSF, Avalon Biomed, USA) is a novel ready-to-use calcium silicate-based endodontic sealer composed of tricalcium/dicalcium silicate and tantalum oxide as a radiopacifier.^6^ NSF demonstrated adequate sealing^7^ and compliance with ISO 6876:2012 standards relative to setting time, flow, radiopacity, and film thickness.^8^ NeoSealer Flo has exhibited ion release and alkaline pH. However, NeoSealer Flo presented solubility above 3%.^8^ The bond strength to dentin and antimicrobial activity of the NSF sealer have not been reported. NeoMTA 2 (NMTA2, Avalon Biomed, USA) is a bioceramic material suitable for application as an endodontic sealer or repair material, depending on the powder-gel ratio.^9^NMTA2 has replaced NeoMTA-Plus with the aim of optimizing flow and filling capacity.^10^ Cytocompatibility, bioactivity, and radiopacity have been described for NMTA.^11^ Furthermore, NMTA2 has demonstrated bond strength values similar to NeoPutty (NuSmile, Houston, United States) and clinical performance in pulpotomy procedures.^12^ There have been no reports in the literature about the antimicrobial potential of NSF and NMTA2.

Bio-C Sealer (BCS, Angelus Industria de Produtos Odontologists S/A, Londrina, Brazil) is a ready-to-use bioceramic sealer.^3^ Satisfactory physicochemical properties,^3^ antimicrobial activity^13^ as well as biocompatibility and bioactive potential^3,14^ have been reported for BCS. However, this sealer has higher solubility than AH Plus (Dentsply DeTrey, Konstanz, Germany).^3,15^ AH Plus Jet (AHP, Dentsply DeTrey, Konstanz, Germany) is an epoxy resin-based sealer in the presentation of paste-paste and supplied in a self-mixing syringe. AHP has shown antimicrobial activity and excellent physicochemical performance. It is often used as a reference for comparing new root canal filling sealers.^16,17^ AHP has antimicrobial activity due to the presence of epoxy resin and amines in its composition.^18^

Considering the constant development of new bioceramic endodontic sealers, it is essential to evaluate their physicochemical, mechanical, and antimicrobial properties to provide support for their clinical use. Therefore, the aim of this study was to evaluate the physicochemical properties, antimicrobial activity against *E. faecalis*, and bond strength of the new bioceramic sealers NSF and NMTA2. AHP and BCS were used for comparisons. The null hypothesis was that there would be no differences among the sealers for all the variables tested.

## Methods

The Experimental Groups are outlined in Table 1. AH Plus Jet was manually mixed by combining equal parts of the two pastes. This method was selected due to its significant impact on the physicochemical and mechanical properties of AH Plus Jet, as was shown in a previous study.^19^

**Table 1:** Materials, manufacturer, composition and proportion used

Materials	Manufacturer	Composition	Proportion
NeoSealer Flo (NSF)	Avalon Biomed, TX, USA	tricalcium silicate, dicalcium silicate, calcium aluminate, aluminum oxide, calcium oxide, tricalcium aluminate, tantalite and calcium sulfate	Ready to use
NeoMTA 2 (NMTA2)	Avalon Biomed, TX, USA	Powder: tricalcium silicate, dicalcium silicate, tantalum oxide, calcium sulfate, tricalcium aluminate.	1 scoop of powder: 3 drops of gel (powder/gel)
		Gel water based	
Bio-C Sealer (BCS)	Angelus, Londrina, PR, Brazil	Calcium silicates, calcium aluminate, calcium oxide, zirconium oxide, iron oxide, silicon dioxide and dispersing agent.	Ready to use
AH Plus Jet (AHP)	Dentsply DeTrey GmbH, Konstanz, Germany	Bisphenol-A Epoxy Resin, Bisphenol-F Epoxy Resin, Calcium Tungstate, Zirconium Oxide, Silica, Benzyldiazines Iron Oxide Pigments, Aminoadamantane, Silicone Oil	1:1 (paste/paste)

### Setting time

Plaster molds were filled with bioceramic sealers were kept immersed for 24 hours, and metal molds of AHP, both 10 mm in diameter and 1 mm in depth, were filled with sealers (n = 6). The setting time (ST) was evaluated in accordance with the standard ISO-6876.^20^ A Gilmore needle type 100 ± 0.5g with an active tip of 2 ± 0.1 mm was used. The materials were kept in an oven at 37ºC and 95% humidity. The ST was determined in minutes (min) as the period between manipulation of the sealer and the time when the Gilmore needle did not produce marks on the surface of materials.

### pH

Polyethylene tubes measuring 10 x 1.6 mm (length x diameter) were filled with sealers (n = 10) and individually immersed in 10 mL of deionized water. The samples were kept in an oven at 37ºC during time intervals of 1, 3, 7, and 14 days after the tubes were immersed. Distilled water was used as Control (C). The measurements were performed in triplicate, using a previously calibrated digital pH meter (Digimed Analytica Ltda., Grupo Digitron analytic, São Paulo, Brazil).

### Solubility

The solubility of the materials was evaluated in accordance with the ANSI/ADA standard, adapted from Carvalho-Junior et al.^21^ Sealer discs measuring 7.75 mm in internal diameter and 1.5 mm high were made, and the tip of a nylon thread (n=6) was included into the sealer mass. After being kept in an oven at 37ºC for 24 hours, the specimens were weighed on a precision scale (Adventurer AR2140; Ohaus, Corporation, Parsippany, USA) to obtain the initial mass. The nylon thread was used to suspend the sealer discs in a plastic flask containing 7.5 mL of distilled water at 37ºC for 7 days without allowing any contact between the sealer and the inner surface of the plastic flasks. The discs were removed from the distilled water, dried with absorbent paper, and inserted into a dehumidifier until the mass stabilized. Solubility was obtained by calculating the mass loss after immersion, expressed as a percentage (%).

### Flow

The test was performed in accordance with the ISO 6876:2012 standard.^20^ A quantity of 0.05 ml of filling sealer (n = 6) was placed on a glass plate. A new glass plate (20 g) was placed over the sealer, and a metal weight of 100 g was applied to the central region of the top plate. After 7 minutes had elapsed, the major and minor diameters of the extent of sealer flow were measured with a digital caliper, in millimeters (mm). When the difference between diameters was less than 1 mm, the average value was recorded.

### Radiopacity

Specimens of each sealer (n = 4), measuring 10 mm in diameter x 1 mm thick were placed on occlusal radiographic films (Insight-Kodak Comp, Rochester, USA) and exposed close to an aluminum scale with variable thickness (2 to 16 mm, in increments of 2 mm). An X-ray apparatus (Instrumentarium Dental, Tomsula, Finland) operating at 60 kV, 7 mA, 0.32 pulses per second, and a focus-film distance of 33 cm was used. The films were developed in a conventional automatic processor (Dent-X 9000; Dent-X, Elmsford, USA). After this, the radiographs were digitized, and the images were transferred to Image Tool 3.0 software (University of Texas Health Science Center at San Antonio, San Antonio, USA). For evaluating the radiopacity of the materials, the area corresponding to each degree on the aluminum scale and the area of sealers were selected. This radiopacity was expressed in terms of the equivalent thickness of aluminum (in mm/Al).

### Bond Strength

Bovine maxillary incisors were selected. After removing the crowns and apical 2 mm, the roots were embedded in laminating resin (Maxi Rubber, São Paulo, Brazil). After the resin had set, the specimens were cross sectioned at a thickness of 2 mm using an Isomet 1000 cutter (Buehler Ltd, Lake Bluff, USA). Root canals were prepared using a standardization protocol with a parallel meter delineator (Bio-Art, São Carlos, Brazil) and a 702 conical trunk carbide drill (Labor Dental, São Paulo, Brazil). Irrigation was performed with 1% sodium hypochlorite (NaOCl). After preparation, the root canals were irrigated with 17% EDTA for 3 minutes, followed by 5 ml of water, and then filled with bioceramic sealers. After 7 days of storage at 37ºC and 95% humidity, the Push-out test was conducted on an Emic DL 2000 machine (Instron, São José dos Pinhais, Brazil) with a load cell of 1 kN operating at a constant speed of 0.5 mm/min and a stainless-steel cylindrical punch with a diameter of 1.2 mm until the material was displaced. The maximum load was recorded in Megapascals (MPa) after entering the height and inner radius of the specimen into the universal testing machine.

### Antibiofilm activity assessment (modified direct contact test)

Hydroxyapatite blocks were contaminated with an inoculum of standard strains of *Enterococcus faecalis* (ATCC 29212) for 7 days. Modified direct contact tests were performed with fresh sealer eluate (50 mg/mL) and sealer after setting for 48 hours (specimen) (n = 6). As a Control Group, distilled water (the test was performed with eluate) and Teflon disc (the test was performed with sealer after setting) were used. After the contact time of 15 hours, the hydroxyapatite blocks with the remaining biofilm were individually placed in microtubes with sterile saline solution, and then serial decimal dilution and plating in triplicate were performed. The plates were incubated at 37ºC for 48 hours. After the incubation time, the colony-forming units were counted (CFU/mL^-1^), and the data were submitted to logarithmic transformation (log^10^).

### Statistical analysis

All data were initially assessed for normality using the Shapiro-Wilk test. Data met the requirements for parametric testing. The physicochemical and antibiofilm data were analyzed using one-way ANOVA to compare groups, followed by post-hoc Tukey tests. A significance level of α = 0.05 was used for all statistical analyses.

## Results

The results are shown in Tables 2, 3, and 4. NMTA2 showed a longer setting time than NSF. BCS had the shortest setting time, and AHP had the longest (p < 0.05). NSF and BCS obtained the highest loss of mass values (p < 0.05) followed by NMTA2 and AHP (p < 0.05). NMTA2 presented higher radiopacity values than NSF and lower values than BCS (p < 0.05). NSF had the lowest radiopacity AHP had the highest values (p < 0.05). BCS showed the largest flow, followed by AHP and NSF (p < 0.05), and NMTA2 showed the smallest flow area (p < 0.05). NSF presented the highest bond strength values, followed by NMTA2, AHP, and BCS (p < 0.05).

**Table 2:** Mean and ± standard deviation of setting time, solubility, radiopacity, flow and bond strength of the sealers evaluated

Variables	AHP	BCS	NSF	NMTA2
Setting time(min)	1253 ± 9.45^a^	148.2 ± 2.48^d^	160.8 ± 3.06^c^	451.8 ± 4.16^b^
Solubility (%)	0.61 ± 0.12^c^	6.04 ± 0.97 ^a^	5.24 ± 0.80^a^	1.89 ± 0.47^b^
Radiopacity (mm/Al)	15.64 ± 0.59^a^	5.57 ± 0.32^b^	3.49 ± 0.17^d^	4.47 ± 0.55^c^
Flow (mm)	21.85 ± 1.62^b^	34.24 ± 1.13^a^	21.72 ± 1.47^b^	14.06 ± 1.01^c^
Bond Strength (MPa)	0.74 ± 0.22^b^	0.53 ± 0.25^b^	3.14 ± 0.54^a^	0.79 ± 0.19^b^

Different letters in the same line indicate statistically significant differences among groups (p < 0.05).

**Table 3:** Mean and ± standard deviation of pH after 1, 3, 7 and 14 days

Varialbe		AHP	BCS	NSF	NMTA2	C
pH	1 day	7.70 ± 0.15^d^	9.4 ± 0.39^c^	10.28 ± 0.03^b^	10.65 ± 0.14^a^	7.63 ± 0.1^d^
	3 days	7.41 ± 0.07^b^	9.84 ± 0.39^a^	9.92 ± 0.18^a^	9.96 ± 0.20^a^	7.22 ± 0.2^b^
	7 days	6.86 ± 0.06^d^	9.60 ± 0.93^bc^	9.82 ± 0.37^ac^	10.41 ± 0.34^a^	7.28 ± 0.0^d^
	14 days	6.71 ± 0.13^d^	9.30 ± 0.78^ac^	8.83 ± 0.73^bc^	9.80 ± 0.48^a^	7.26 ± 0.2^d^

Different letters in the same line indicate statistically significant differences among groups (p < 0.05).

**Table 4:** Antibiofilm activity – CFU count mL-1 log10

Groups	NSF	BCS	AHP	NMTA2	C+
Setting 48h	5.47 ± 0.29^b^	5.48 ± 0.26^b^	5.95 ± 0.07^b^	4.56 ± 0.22^d^	6.61 ± 0.28 ^a^
Sealer eluate	1.21 ± 1.33^c^	1.31 ± 1.44^c^	0.73 ± 1.12^c^	3.19 ± 0.56^b^	7.27 ± 0.32^a^

Different letters in the same line indicate statistically significant differences among groups (p < 0.05).

NSF, BCS, and NMTA2 presented alkaline pH in all time intervals evaluated. NMTA2 presented the highest values in all time intervals. AHP presented pH values similar to those of the control group, in all time intervals (p > 0.05).

Antibiofilm activity was observed for all materials, in both tests, in comparison with the positive control (C+). A higher level of antibiofilm activity was observed with fresh sealer eluate for all sealers. At 48h after setting, NMTA2 showed the highest level of antibiofilm activity (p < 0.05), followed by NSF, BCS, and AHP (p < 0.05). For the test with fresh sealer eluate, NSF, BCS, and AHP showed higher levels of antibiofilm activity, compared with NMTA2 and the Control (p < 0.05).

## Discussion

The aim of the study was to evaluate the physicochemical, antimicrobial properties, and bond strength of ready-to-use bioceramic sealer NSF and powder-gel NMTA2 in comparison with BCS and AHP. Differences between the material properties led to the rejection of the null hypothesis.

All the bioceramic sealers showed a setting time in accordance with recommended by ISO 6876:2012 (30 min to 72h). However, AHP had the longest setting time in comparison with all the bioceramic sealers. AHP also had a longer setting time than the ready-to-use root canal sealers BCS and BCS Ion^+^.^22^ The composition of calcium silicate-based sealers can influence the setting time.^22^ Moreover, the particle size, temperature, and environmental humidity also affected the setting time of bioceramic sealers.^23,24^These factors may explain the variation in setting time observed in the present study.

According to the ISO 6876/2012 standards, the solubility of endodontic sealers must not exceed 3% loss of mass after immersion in distilled water for 24 hours.^20^ However, longer periods of evaluation can be used.^3^ In this study, NSF and BCS showed solubility higher than those established by ISO standards, corroborating previous investigations.^8,3,25^ In contrast, low solubility values were observed for AHP followed by NMTA2 bioceramic sealer. The quantity of tricalcium and dicalcium silicate and calcium aluminate present in bioceramic materials can influence the change in mass of these materials.^26^ Furthermore, the results may be related to the greater availability of liquid in the hydration reaction of bioceramic sealers in the powder-liquid composition in comparison with ready-to-use sealers.^22^ The hydration reaction has a direct effect on the adequate physicochemical and biological performance of calcium silicate-based sealers.^22^ Solubility is closely related to the alkalization capacity of bioceramic materials.^26^ In this study, all calcium silicate-based sealers showed alkaline pH in all the evaluation time intervals. These findings are justified by the capacity of bioceramic sealers to release calcium and hydroxyl ions, which can play an important role in the repair of periapical tissues by the formation of mineralized tissue.^27^

When the radiopacity was evaluated, our results showed that all sealers were in accordance with ISO 6876 standards (> 3 mm Al).^20^ However, AHP presented higher radiopacity, followed by BCS, NMTA2, and NSF. These differences could be attributed to the quantities and proportions of each radiopacifying agent in the sealers evaluated.^8,28^ AHP contains zirconium oxide and calcium tungstate as radiopacifiers that promote radiopacity.^28^ In addition to adequate radiopacity, bioceramic sealers must present flow in accordance with ISO standards, allowing the filling of irregularities in root canals.^3^ In this study, NSF, BCS, and AHP presented flow in accordance with ISO 6876 standards, corroborating previous studies.^29^ However, NMTA2 showed slightly lower flow (14.06 mm) than recommended by ISO (≥ 17 mm). Although no other study has evaluated the flow of NMTA2, its chemical composition is similar to NeoMTA Plus, making correlations possible. A previous study showed a flow of 13.98 mm for NeoMTA Plus when using the conventional ISO test,^30^ similar to this study. However, it is important to emphasize that flow assessment using conventional ISO methodology may not show a correlation with the filling capacity of bioceramic materials.^30,31^ Adequate filling capacity in curved mesial canals of mandibular molars was reported for NeoMTA Plus in micro-computed tomography (micro-CT) evaluations, demonstrating similarity to AH Plus.^30^

Bioceramic-based sealers must present bond strength to dentin to promote proper interface between the filling material and the root canal walls.^32^ The push-out test of this study showed that NSF had the highest bond strength, higher than NMTA2, BCS, and AHP. Although this is the first study to evaluate the bond strength of NSF, our results corroborate a previous study that demonstrated higher bond strength for the ready-to-use sealer in comparison with AHP.^33^ Further studies are necessary to evaluate the bond strength of bioceramic sealers in powder-liquid or ready-to-use form.

Antimicrobial activity is an essential property for endodontic sealers since it contributes to the control of residual infection.^3^ *E. faecalis* is a facultative anaerobic gram-positive bacterium, commonly associated with endodontic treatment failure and it has great potential for the formation of resistant biofilm.^32^ The present findings indicated antimicrobial activity for all sealers when using fresh sealer eluate in comparison with the 48-h setting materials. These results may be related to the formation of calcium hydroxide during the hydration reaction of bioceramic sealers, which promotes an alkalizing effect and causes damage to the bacterial membrane and DNA.^27^ NMTA2 exhibited lower level of antibiofilm activity in the fresh sealer eluate and a higher level of activity after 48 hours of sealer hardening. These results may be associated with the prolonged setting time of calcium silicate-based endodontic sealers.^27,22^

## Conclusion

NeoSealer Flo and NeoMTA 2 exhibit bond strength and the required physicochemical properties, including setting time, pH, and radiopacity, suitable for clinical applications. Additionally, NeoMTA 2 shows solubility according to ISO 6876 standards. Both sealers demonstrate antibiofilm activity against *E. faecalis*. However, NeoSealer Flo shows higher solubility, and NeoMTA2 exhibits lower flow rates than the values recommended by ISO.

## Data Availability

After publication the data will be available on demand to the authors - condition justified in the manuscript.
